# Anti-Lung-Cancer Activity and Liposome-Based Delivery Systems of ****β****-Elemene

**DOI:** 10.1155/2012/259523

**Published:** 2012-11-28

**Authors:** Meiwan Chen, Jinming Zhang, Siqin Yu, Shengpeng Wang, Zaijun Zhang, Jianqiang Chen, Jian Xiao, Yitao Wang

**Affiliations:** ^1^State Key Laboratory of Quality Research in Chinese Medicine, Institute of Chinese Medical Sciences, University of Macau, Macau 999078, China; ^2^Beijing DDS-Venturepharm .T. Corp, Beijing 100097, China; ^3^Institute of New Drug Research, Guangdong Province Key Laboratory of Pharmacodynamic, Constituents of Traditional Chinese Medicine & New Drug Research, College of Pharmacy, Jinan University, Guangdong 510632, China; ^4^School of Pharmacy, Cixi Hospital, Wenzhou Medical College, Wenzhou 325035, China

## Abstract

In the past decade, **β**-elemene played an important role in enhancing the effects of many anticancer drugs and was widely used in the treatment of different kinds of malignancies and in reducing the side effects of chemotherapy. Further study showed that it is also a promising anti-lung cancer drug. However, the clinical application of **β**-elemene was limited by its hydrophobic property, poor stability, and low bioavailability. With the development of new excipients and novel technologies, plenty of novel formulations of **β**-elemene have improved dramatically, which provide a positive perspective in terms of clinical application for **β**-elemene. Liposome as a drug delivery system shows great advantages over traditional formulations for **β**-elemene. In this paper, we summarize the advanced progress being made in anti-lung cancer activity and the new liposomes delivery systems of **β**-elemene. This advancement is expected to improve the level of pharmacy research and provide a stronger scientific foundation for further study on **β**-elemene.

## 1. Introduction

As the leading cause of mortality in the United States with 556,500 deaths, lung cancer leads up to 163.700 deaths exceeding the sum of death caused by cancers of breast, genital system, and urinary system according to cancer statistic data in 2011 [1]. Both over one million deaths and dramatically growing number of new case of lung tumors worldwide annually turn lung cancer into an epidemic disease [2]. Surgical resection is the best treatment of lung cancer, but over 75% of patients would like to choose chemotherapy due to the development of metastases [3]. Platinum-based combinations have been the standard first-line chemotherapy all the time in clinic, while current diagnosis for most patients is not satisfactory with overall survival at 5 years of only 10–15% [4, 5]. In addition, taxanes, wildly used clinically for lung cancer patients in recent years, also could result in common side effects such as neutropenia, alopecia, allergic reaction, neuropathy, and fatigue, being similar with other antimitotic agents [6]. To screen and develop a novel effective candidate drug as the combination of substantial anticancer drugs would decrease overlapping side effect profiles and overcome the resistance of tumor cells to some extent [7], vastly promoting the lung cancer chemotherapy.

**Figure 1:**
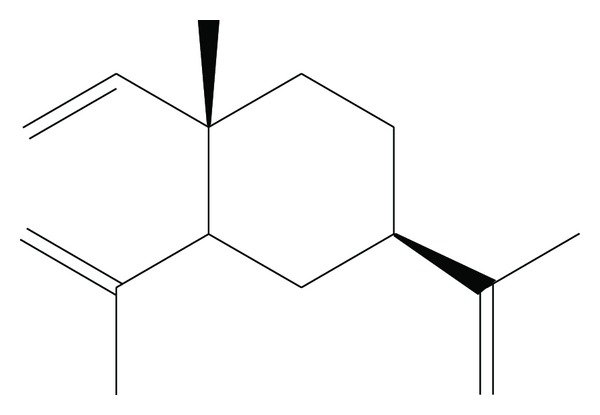
Chemical structure of **β**-Elemene.

*β*-elemene, isolated first from Zingiberaceae *Curcuma wenyujin* rhizome (*Curcuma wenyujin* Y. H. Chen et C. Ling) and existing in excess of 50 Chinese herbs and plants, is an effective sesquiterpene vinyl monomer with the structure of 1-methyl-1-vinyl-2,4-diisopropenyl-cyclohexane (Figure 1) [8, 9]. *β*-elemene has shown direct and indirect substantial suppression effects to broad spectrum tumor via *in vivo* and *in vitro* tests, such as inducing tumor cell apoptosis, inhibiting tumor cell proliferation, antitumor metastasis, and protecting active immunity [10, 11]. Meanwhile, fewer adverse effects and lower cost also benefit *β*-elemene to have a wide application prospect. There have been some side effect reports of *β*-elemene about slight fever, gastrointestinal reactions, and local pain [12], instead of bone marrow, liver, cardiac toxicities so far. In clinical application, *β*-elemene, used alone or combined with other chemotherapeutics against various tumors, like lung cancer, gastrointestinal cancer, breast cancer, and other tumor diseases, at the same time enhances the immunity of the patients, with remarkable therapy effects [9, 13]. Recently, pharmacological studies [13–15] also have reported that *β*-elemene would be effective to some hyperplasic and proliferative disorders such as prostatic hypertrophy, lung tumor, ovarian tumour, and melanoma. 

Owing to these bioactivities above, it seems to find the spring of cancer chemotherapy for pharmaceutics by means of *β*-elemene on a large scale. Some formulations of *β*-elemene including injection, emulsion injection, freeze-dried powder, and aerosol have been applied. Injectable emulsion of *β*-elemene has been developed and launched clinic use on the therapy of cancers in China since 1995 [16, 17]. Afterwards, an injection preparation containing 85%  **β**-elemene developed by CSPC Pharmaceutical Limited Cooperation (China) has been approved by the SFDA, China, for the treatment of brain tumours and other carcinomas [18]. Unfortunately, *β*-elemene, greatly suffered from poor water solubility, stability, and low bioavailability, leads to a relatively low bioavailability with oral administration, which means lots of solubilizing excipients are needed to prepare injections as well. Yet, with the growing amounts excipients, the increase of dosage and adverse reactions, especially the blood vessels stimulation caused by the instability of drug and excipients [19], produces a lot of inconvenience to patients and restricts its clinical applications likewise. Therefore, there is an urgent need to study alternative delivery system for *β*-elemene. 

In recent years, the research of novel delivery systems for *β*-elemene has been extremely active and made a lot of advancements including microemulsion, microcapsules, liposomes, solid lipid nanoparticle, and more. Among the different strategies employed to improve the pharmacokinetics of poorly soluble drug like *β*-elemene, liposome-based delivery system shows excellent delivery property to enhance permeability and retention. Liposome, with the similar structure of biomembrane composing of outer phospholipid bilayer and inner hollow vesicle, leads to a more rapid and complete absorption, liposomal encapsulation, and a degree of “passive” or “physiological” targeting to tumor tissue [20]. It has aroused researchers great interest since 1971 when Rymen proposed the liposome as a novel drug delivery system [21] and showed significant advantages [22]. For example, it can enhance drug stability, reduce toxicity, and achieve target direction action, lymphatic directionality, and long-term sustained release. 

Based on the significant antitumour activity and a low level of toxicity represented above, to develop *β*-elemene as a chemotherapeutic agent would be a promising approach in prospective in-depth study. In this paper, we firstly overviewed the anti-lung cancer pharmacological action and mechanism of investigation of *β*-elemene in recent years. Then, we discussed research progress in liposome-based delivery system of *β*-elemene for anti-lung cancer.

## 2. Anti-Lung Cancer Activity

According to the phase II clinical trials of *β*-elemene currently ongoing in USA, *β*-elemene has obtained more and more attention to investigate its anti-lung cancer activity and effect mechanism *in vivo* and *in vitro* recently. Herein, the experimental studies on both single use and combination with other agents will be represented.

For the reason [23] that about 85% of lung cancer patients are diagnosed as non-small-cell lung cancer (NSCLC), the 1-year survival rate of which is only 10–15%, most of studies focused on various NSCLC models. Wang et al. [24] uncovered the effect of *β*-elemene on G2-M arrest in NSCLC cells, which was mediated partially by a Chk2-dependent mechanism, and NSCLC cells apoptosis promotion would be the mechanism to inhibit NSCLC, using human cell lines H460 and A549. Differing from human lung fibroblast CCD-19Lu cell line and bronchial epithelial NL20 cell line, NSCLC cells showed a mitochondrial release of the cytochrome c-mediated apoptotic pathway induced by *β*-elemene. This view was consistent with another further study [25] that human NSCLC cell lines A549 apoptosis induced by *β*-elemene would be related to downregulate antiapoptotic proteins Bcl-2 and inhibit the PI3 K/Akt/mTOR pathway associated to cell growth and cell cycle progress promotion. Similar to A549 cells, Li et al. [26] observed that *β*-elemene can mediate Cbl-regulated Akt and ERK activation to induce cell apoptosis in lung cancer cells. Since radiotherapy is still the most important therapeutic way for lung cancer, radiosensitivity of *β*-elemene observed recently also arouse researchers' interests. Apart from promoting the tumor cell apoptosis directly, *β*-elemene showed its radiosensitization effect to lung adenocarcinoma A549 cell by upregulating P53 gene expression and inhibiting the Bcl-2 gene, inducing apoptosis indirectly [27]. Li et al. [28] conducted an experiment to investigate the underlying mechanism of radiosensitivity enhancement of *β*-elemene to lung adenocarcinoma tumor. Using female athymic BALB/c nu/nu mice to establish lung tumor models, it was showed that *β*-elemene (45 mg/kg) had the potential to decrease significantly surviving and hypoxia-inducible factor HIF-1a protein's radiation, which would be novel targets of *β*-elemene.

Except the single use for lung cancer, most of the time *β*-elemene was used to combine with some first-line anticancer agents like taxol. Thus, to investigate the therapeutic effect and safety, some researchers focused on studying the combination form of *β*-elemene and some chemotherapeutic agents. Synergistic antitumor effect of *β*-elemene (20 or 50 *μ*g/mL) and etoposide (15 *μ*g/mL) to A549 NSCLC has been observed by Zhang et al. [29], which was mediated by the cleavage of PARP, the upregulation of Bax, p53 and p21, as well as the suppression of cyclin D1. In addition, studies [30] have shown that *β*-elemene could eliminate the resistance of NSCLS cells to Gefitinib. That would indicate a combination of *β*-elemene and Gefitinib could remarkably improve the sensitivity to the Gefitinib-resistant cell line by modulating the levels of cell cycle checkpoint protein p21 and inhibiting cdk2/4 and cyclinE/D1 activation. Zhao and coworkers [31] found the synergistic interactions between *β*-elemene and taxanes, showing enhancing tumor cell apoptosis, suppressing specific “survival” gene expression, and enhancing cellular uptake. Data indicated that the combination *β*-elemene with taxanes can increase the cytochrome C released from mitochondria, significant caspase-8 and -3 cleavage, and downregulation of Bcl-2 and -X_L_ expression, which is a p53- and Fas-independent pathway via mitochondria in human lung cancer cells. Meanwhile, the fact that *β*-elemene can change the permeability of plasmalemma and increase the concentration of taxanes into the cell to inhibit human lung cancer cell growth and synergies also could be observed from this data. Some observations from Li et al. [23] suggested that *β*-elemene sensitized NSCLC cells to cisplatin via a mitochondria-mediated intrinsic apoptosis pathway involving Bcl-2 family proteins and IAPs (inhibitor of apoptosis proteins). These results might provide an effective certification to improve the antitumor action of chemotherapy agents.

## 3. Liposome-Based Delivery Systems of *β*-Elemene

As a potential drug delivery system, liposome is gaining more and more attention to become a new type of targeted antitumor effective route for *β*-elemene in order to overcome some problems in current formulation. In present paper, the introduction about liposome preparations of *β*-elemene including preparation method, quantitative determination method, pharmacokinetic behaviour, and modified liposome will be represented, respectively, as follows.

In the preparation process of liposome, there are basically similar ingredient materials containing drugs, phospholipid for skeleton, and cholesterol as buffers to regulate liquidity of phospholipid bilayer. Nevertheless, what is important to affect the quality of liposome would be the preparation methods, such as thin-film hydration (TFH), reverse evaporation (REV), microemulsion [32], remote loading [33], supercritical reverse phase evaporation [34], and supercritical reverse phase evaporation [35]. Song et al. [36] prepared *β*-elemene liposome by film dispersion method. Huang et al. [37] reported that REV with high-pressure extrusion method would be most appropriate to produce *β*-elemene liposome for the reason of smaller diameter, uniform distribution, higher drug loading, and encapsulation efficiency, comparing TFH, REV, and REV with high-pressure extrusion. Similarly, Du and coworkers [38] prepared *β*-elemene liposome with TFH method by the combination of orthogonal design to optimize the amounts of lecithin and cholesterol, as well as ultrasonic entrapment time. Additionally, some researchers develop the modified liposome to broaden its use and decrease adverse effects. To decrease the irritation to blood vessel and avoid the premature drug leakage of liposome via oral administration in digestive system, Zhang et al. [39] complexed *β*-elemene liposome with polyvinylpyrrolidones (PVPs) as a three-dimensional protective agent in its surface via ethanol inpouring method, unchanging its partial size and entrapment efficiency significantly. To extend the retention time *in vivo*, PEG2000-completed long-circulating *β*-elemene liposomes with high encapsulation efficiency and good stability were developed via the ethanol injection technique [40]. 

The amount of loading drug in liposome and the entrapment efficiency of liposome will be evaluated by means of the content determination of *β*-elemene in liposomes generally via high performance liquid chromatography (HPLC) [41], and gas chromatography (GC) [42] follows the preparation process. The reversed phase HPLC method was established by Qi and Liang [43] to determine the content of *β*-elemene liposomes, which was simple, rapid, and sensitive with good separation efficiency then after contents. Xie et al. [44] established a GC method to determine the content and entrapment efficiency of *β*-elemene liposomes, using ultra-filtration to separate free drug from *β*-elemene liposomes. They found that the method was accurate, reliable, simple, and fast. Wang et al. [45] reported they separated the free *β*-elemene with minicolumn centrifugation method and determined the entrapment efficiency of *β*-elemene liposomes with GC.

Pharmacokinetics research, behind the physicochemical evaluation like drug loading rate, also plays an important role in assessing the advantage of liposomes drug delivery system. Song et al. [46] compared tissue distribution of *β*-elemene in rats after intravenous injection of *β*-elemene liposomes and *β*-elemene injection from market, respectively, which represented the result that more amounts of *β*-elemene detected in heart, spleen, and kidney tissues using *β*-elemene liposomes. To some extent, it means liposome would be a better drug delivery property. In order to evaluate the better targeting and controlled-release characters of *β*-elemene liposome, the increasing trend of both area under the curve (AUC) and mean residence time (MRT), as well as statistically remarkable increase of peak concentration (*C*_max⁡_) of *β*-elemene in rat blood after using *β*-elemene liposome compared to that of common *β*-elemene injection from market, has been observed by Wu et al. [47]. Moreover, based on the PVP-coated *β*-elemene liposome for oral administration, Zhang et al. [48] compared its pharmacokinetic parameters with that of common *β*-elemene oral emulsion from market to investigate whether the bioavailability has been improved. The results did make clear that ordinary oral emulsions, as the reference formulation, has the lower bioavailability than PVP-coated *β*-elemene liposome, with the relative bioavailability being (140.2 ± 7.5)%.

## 4. Conclusions

In short, *β*-elemene, being a potential anticancer drug, has a broad application foreground for anti-lung cancer mainly because of its three advantages as follows: (1) considerable amounts and abundant sources from over 50 kinds of herbs and plants; (2) efficient anti-cancer effects including inhibiting lung cancer cells' angiogenesis, inducing tumor cell apoptosis, enhancing radiosensitivity and favourable chemo-therapeutic effects in combination of other anticancer agents; (3) slight side-effects, such as little damages to the liver and kidney function or no bone marrow suppression. Since it is worth developing and the limitation of its existing preparations, it would be the time to develop the novel drug delivery system for *β*-elemene, which is fairly represented by liposome-based delivery systems. So much work about *β*-elemene liposome has been done, while to exploit the liposome-based delivery systems with the properties of targeting function of drugs increase, stability improvement and sustained and controlled release is still meaningful. With the development and penetration of basic and clinical research including network pharmacology, system biology, and clinical trials, which provide reasonable evidence to anti-lung cancer activity of *β*-elemene, increasing molecular pharmaceutical technologies makes *β*-elemene liposome-based delivery system possible. Undoubtedly, development of *β*-elemene liposome with better pharmacological effects and optimized technologies will be critical for future applications in anti-lung cancer of *β*-elemene clinically.
